# Untargeted metabolomics unveiled the role of butanoate metabolism in the development of *Pseudomonas aeruginosa* hypoxic biofilm

**DOI:** 10.3389/fcimb.2024.1346813

**Published:** 2024-02-16

**Authors:** Ahmed G. Abdelhamid, Ahmed E. Yousef

**Affiliations:** ^1^ Department of Food Science and Technology, The Ohio State University, Columbus, OH, United States; ^2^ Botany and Microbiology Department, Faculty of Science, Benha University, Benha, Egypt; ^3^ Department of Microbiology, The Ohio State University, Columbus, OH, United States

**Keywords:** bacterial biofilm, *Pseudomonas aeruginosa*, metabolomics, metabolic markers, *in vitro* biofilm, butanoate metabolism, acetoin, hypoxic biofilm

## Abstract

*Pseudomonas aeruginosa* is a versatile opportunistic pathogen which causes a variety of acute and chronic human infections, some of which are associated with the biofilm phenotype of the pathogen. We hypothesize that defining the intracellular metabolome of biofilm cells, compared to that of planktonic cells, will elucidate the metabolic pathways and biomarkers indicative of biofilm inception. Disc-shaped stainless-steel coupons (12.7 mm diameter) were employed as a surface for static biofilm establishment. Each disc was immersed in a well, of a 24-well microtiter plate, containing a 1-mL Lysogeny broth (LB) suspension of *P. aeruginosa* ATCC 9027, a strain known for its biofilm prolificacy. This setup underwent oxygen-depleted incubation at 37°C for 24 hours to yield hypoxic biofilms and the co-existing static planktonic cells. In parallel, another planktonic phenotype of ATCC 9027 was produced in LB under shaking (200 rpm) incubation at 37°C for 24 hours. Planktonic and biofilm cells were harvested, and the intracellular metabolites were subjected to global untargeted metabolomic analysis using LC-MS technology, where small metabolites (below 1.5 kDa) were selected. Data analysis showed the presence of 324 metabolites that differed (*p <* 0.05) in abundance between planktonic and biofilm cells, whereas 70 metabolites did not vary between these phenotypes (*p* > 0.05). Correlation, principal components, and partial least square discriminant analyses proved that the biofilm metabolome is distinctly clustered away from that of the two planktonic phenotypes. Based on the functional enrichment analysis, arginine and proline metabolism were enriched in planktonic cells, but butanoate metabolism was enriched in biofilm cells. Key differential metabolites within the butanoate pathway included acetoacetate, 2,3-butandiol, diacetyl, and acetoin, which were highly upregulated in the biofilm compared to the planktonic cells. Exogenous supplementation of acetoin (2 mM), a critical metabolite in butanoate metabolism, augmented biofilm mass, increased the structural integrity and thickness of the biofilm, and maintained the intracellular redox potential by balancing NADH/NAD^+^ ratio. In conclusion, *P. aeruginosa* hypoxic biofilm has a specialized metabolic landscape, and butanoate pathway is a metabolic preference and possibly required for promoting planktonic cells to the biofilm state. The butanoate pathway metabolites, particularly acetoin, could serve as markers for biofilm development.

## 1 Introduction

*Pseudomonas aeruginosa* is an opportunistic pathogen that exhibits considerable resistance to antibiotics and poses a significant health risk, particularly to immunocompromised individuals. The pathogen is implicated in both acute and chronic infections in patients suffering from conditions such as cancer, traumas, cystic fibrosis, chronic obstructive pulmonary disease, and ventilator-associated pneumonia (Rossi et al., 2020; Cendra and Torrents, 2021; Jurado-Martín et al., 2021). This organism is associated with substantial mortality rates, contributing to 12.9% of deaths from antibiotic-resistant bacterial infections in Europe (Botelho et al., 2019), and causing 7.1% of healthcare-associated infections in the United States (McCarthy and Paterson, 2017). Two distinct phenotypes of *P. aeruginosa* are involved in disease pathogenesis. Acute infections (e.g., ventilator-associated pneumonia) are caused by planktonic cells expressing essential virulence factors, whereas chronic infections are caused by a biofilm phenotype of the bacterium (Bomberger et al., 2009).

In the biofilm state, survivability of *P. aeruginosa* is enhanced by its ability to persist in low-oxygen (hypoxic or anoxic) environments, which are often present within the host tissues or secretions during an infection (Qin et al., 2022). To adapt to such unfavorable conditions, a sub-population of *P. aeruginosa* deploys a survival strategy by transitioning into a particular pathoadaptive phenotype. This phenotypic shift is characterized by expression of antibiotic efflux proteins, acquisition of adaptive genetic mutations, and a loss of motility. Concurrently, these adapted cells produce exopolysaccharide matrix (Wu, 2019), in conjunction with transition to a biofilm mode of living. Microaerophilic and anaerobic environments, found within human body niches (e.g., cystic fibrosis airways), facilitate robust biofilm formation by *P. aeruginosa* (Rossi et al., 2020). Considering its significance, oxygen-depleted condition was implemented in the current study for the development of *P. aeruginosa* biofilm.

*P. aeruginosa* has a remarkable metabolic versatility with expansive suite of catabolic and anabolic genes that enable the microorganism to thrive in hostile niches (La Rosa et al., 2018; Crabbé et al., 2019). However, a comprehensive understanding of the metabolic profile of *P. aeruginosa* biofilms remains elusive. Gaps in knowledge remain, particularly in relevance to the metabolites that uniquely differentiate biofilm cells from their planktonic counterparts. Researchers have used planktonic cells grown under shaking conditions as a physiological benchmark to compare the changes associated with the biofilm state (Leggett et al., 2022a), but the metabolic divergence between static biofilm cells and the co-existing planktonic cells remains less explored. The latter, which do not adhere to surfaces to form biofilms, may exhibit a distinct metabolic behavior that is yet to be elucidated. Addressing this gap, the current study compares the metabolic behavior of hypoxic *P. aeruginosa* biofilms to planktonic cells that were cultivated under aerobic (shaking) or oxygen-depleted static conditions. Therefore, the study was conducted to (1) decipher the differential metabolomic characteristics of *P. aeruginosa* in the biofilm state, compared to its planktonic counterpart, under oxygen-depleted conditions, and (2) identify the specific metabolic pathways and associated metabolites that were enriched and necessary for the development of *P. aeruginosa* biofilms. These metabolites may hold a potential as biomarkers for this bacterium’s biofilm state.

## 2 Material and methods

### 2.1 Bacterial strains and culture conditions

*P. aeruginosa* ATCC 9027 was obtained from the culture collection of the Food Microbiology Laboratory at The Ohio State University (Columbus, OH, USA). The strain has been shown to be a prolific biofilm producer (Di Onofrio et al., 2019; Zhou et al., 2019; Kamarajan and Muthusamy, 2020). The *P. aeruginosa* strain was grown in Lysogeny broth (LB; Fisher Scientific, Fair Lawn, NJ, USA) at 37°C with shaking at 200 rpm for 24 hours until the bacterium population reaches ~ 9 log CFU/mL.

### 2.2 Planktonic and biofilm modes of growth

Planktonic cells, grown under shaking or static conditions, were investigated. For planktonic cells grown under shaking (designated as PLSH cells), *P. aeruginosa* ATCC 9027 overnight culture was diluted 1:100 in LB (population is approximately 7 log CFU/mL) and incubated at 37°C with shaking at 200 rpm for 24 h. For biofilm development, stainless steel coupons (12.7 mm diameter, 304 stainless-steel disc coupons: Fisher Scientific) were used; one coupon per well of a 24-well microtiter plate (Corning™ Costar™; Fisher Scientific). One-mL aliquot of the 1:100–diluted overnight culture of *P. aeruginosa* ATCC 9027 was dispensed in each well. The microtiter plate was statically incubated at 37°C for 24h under oxygen-depleted conditions using a closed jar containing anaerobic pouches (BD GasPak™ EZ system; Becton, Dickinson and Company, Spark, MD, USA). Biofilm cells were harvested as detailed later. The cultured LB medium surrounding the coupon within the microtiter plate wells were gently aspirated; this cell suspension contains the planktonic cells that did not attach to the coupon surface, and these cells were designated as planktonic cells under static conditions (PLST cells).

### 2.3 Preparation of samples for metabolomic analysis

For aerobic planktonic *P. aeruginosa* (PLSH), cells were harvested by centrifugation at 12,900 x *g* for 8 min at 4°C. The cell pellet was washed twice using 1 mL phosphate-buffered saline (PBS, pH 7.2; Fisher Scientific). Similarly, PLST cells were harvested and washed twice using PBS. Biofilm cells were harvested from the surface of the stainless-steel coupons as follows. After PLST cells aspiration, the coupons were washed twice by adding 1 mL PBS to each well for removing any remaining PLST cells or residual LB medium. After washing, each biofilm-carrying coupon was transferred to a 50-mL Falcon tube containing 3 mL of PBS. The biofilm mass was scraped from the surfaces of the coupon into the PBS solution using a sterile spatula prior to vortexing the tube content for 2-3 min to release any remaining adherent biofilm cells. The biofilm cells in the resulting suspension were harvested by centrifugation as described earlier. Immediately after harvesting, pellets (~ 8 log CFU) representing the planktonic (PLSH and PLST) and biofilm cells were individually resuspended in 600 µL of cold methanol (Fisher Scientific)-deionized water mixture (1:1 ratio) in 1.5-mL microcentrifuge tubes (Eppendorf, Enfield, CT, USA) containing 300 mg of a mixture of 0.1-mm and 0.5-mm glass beads (BioSpec Products, Inc., Bartlesville, OK, USA). To release the intracellular metabolites, cells were homogenized (Mini Bead Mill Homogenizer; VWR, Chicago, IL, USA) for 9 min at 1.5–min intervals at a speed of 5 m/s. After homogenization, an additional 500 µL of methanol-deionized water mixture (1:1 ratio) were added to the homogenized samples, and the mixture was centrifuged at 12,900 x *g* for 10 min at 4°C to sediment the cells debris and the glass beads. Supernatants were collected and dispensed into glass tubes before adding 6 mL of HPLC-grade methanol/water/chloroform mixture (1:1:1 ratio). The tube mixtures were centrifuged at 4,300 x *g* for 10 min at 4°C to allow phase separation. The top aqueous phase was collected, and any residual methanol was evaporated using a vacuum concentrator (Automatic Environmental SpeedVac^®^ System; Savant, Hyannis, MA, USA) to produce a solvent–free water extract containing the intracellular metabolome, which was used for the untargeted metabolomic analysis using liquid chromatography-mass spectrometry (LC-MS) technique.

### 2.4 LC-MS–based metabolomic analysis

Aliquots (5 µL) of the metabolomic biofilm and planktonic samples were analyzed by ultra-high pressure LC equipment using low-coverage C18 phase column (Waters XSelect HSS T3 column; Waters Corporation, Milford, MA, USA) with a solvent gradient program as follows: 5–95% methanol with 0.1% formic acid (mobile phase-B) for 50 minutes at a flow rate of 0.2 mL/minute. The column temperature was maintained at 40°C. Mobile-phase-A was water with 0.1% formic acid and mobile phase-B was methanol with 0.1% formic acid. Chromatographic fractions were injected into a high-resolution mass spectrometer (ThermoScientific Q-Exactive Plus system; ThermoFisher Scientific) equipped with an electrospray ionization (ESI) source in both negative and positive ion-modes with an ESI voltage of 5500 V for positive mode and 4500 V for negative mode; nebulizer gas (air), turbo gas (air), and curtain gas (nitrogen) were set at 50, 50 and 25 psi, respectively. Metabolomic extracts from the three phenotypes were performed in three repeats and each repeat was injected and analyzed twice in the LC-MS system. The Non-linear Dynamics Progenesis QI software (Waters Corporation) was used for data acquisition and preprocessing. Peaks with intensities above the background were included in the metabolomic data analysis.

### 2.5 Effect of acetoin on *Pseudomonas aeruginosa* biofilm using *in vitro* assay

Acetoin, a key metabolite in the butanoate pathway, was tested experimentally at different non-inhibitory concentrations (0.0, 0.25, 0.5, 1.0, and 2.0 mM) to evaluate its effect on *P. aeruginosa* ATCC 9027 biofilm mass either at the early stage of biofilm formation (attachment) or at a later stage of biofilm cycle (maturation). To assess the impact of acetoin on the early stage of biofilm formation, a standard 96-well microtiter biofilm assay (Coffey and Anderson, 2014) was used with modifications. For quantitative measurements of *Pseudomonas* biofilm mass in the presence or absence of acetoin, crystal violet was used to stain the adhering biofilm on the surface of the microtiter wells. Briefly, *P. aeruginosa* cells were grown in LB broth for 24 h at 37°C with shaking at 190 rpm. After incubation, the cultured *P. aeruginosa* cells were diluted 1:100 in fresh LB broth and 150-µL aliquots of the diluted culture were distributed in triplicates, with or without acetoin treatment, in the 96-well microtiter plate. Blank wells included uninoculated fresh LB broth. The microtiter plates were covered with parafilm and incubated in anaerobic jars for 24 h at 37°C without shaking. After incubation, planktonic cells were aspirated and discarded, the microtiter plate wells were washed three times with deionized water to remove remaining planktonic cells or media components. After washing, the microtiter plate was dried for 25 min at 55°C, and wells were stained with 150 µL of 0.1% crystal violet solution for 25 min at room temperature. Subsequently, excess stain was removed, and the plates were washed with deionized water until the blank wells showed no stain. Crystal violet-stained biofilms were solubilized with 95% ethanol for 1 hour and biofilm mass was inferred as the optical density (OD) determined at 590 nm.

To investigate the impact of acetoin on biofilm maturation (i.e., following initial attachment), the standard microtiter biofilm assay was modified as follows. Acetoin was introduced, at predetermined concentrations, to the 96-well plates containing a pre-grown 8-hour culture of *P. aeruginosa* by which time the cells were anticipated to have adhered to well surfaces.

### 2.6 Effect of acetoin on *Pseudomonas aeruginosa* biofilm structure using live cell fluorescence microscopy

To visualize the impact of acetoin on *P. aeruginosa* biofilm structure, cells were grown in LB broth at 37°C and 190 rpm for 24 h, as previously outlined. These cultures were then diluted 1:100 in fresh LB broth and one milliliter of the diluted culture was aliquoted in duplicate into a four-well chamber slide system (Nunc™ Lab-Tek™ II Chamber Slide™ System; ThermoFisher Scientific), which, according to the manufacturer, is ideal for live cell imaging and consistent cell growth and adherence to surfaces. Each well was either treated with 2 mM acetoin or left untreated as a control. The slide system was then incubated in an anaerobic jar at 37°C for 24 h. Post-incubation, planktonic cells were gently aspirated, and the adhering biofilm was rinsed three times with sterile water to eliminate residual media and unattached cells. The adherent biofilm was subsequently stained with a green-fluorescent nucleic acid stain (SYTO™ BC; ThermoFisher Scientific) for 15 minutes in a dark setting. Afterwards, the excess stain was removed through further washing. Biofilms, both acetoin-treated and untreated, were visualized using an inverted confocal microscope (Nikon A1R ECLIPSE Ti‐E; Nikon Instruments Inc., Melville, NY, USA).

### 2.7 Effect of acetoin on the redox state of biofilm cells

To gain a mechanistic insight into the role of acetoin in increasing biofilm formation, the redox state of biofilm and planktonic cells (PLST) of *P. aeruginosa* was measured by determining the intracellular levels of oxidized and reduced forms of nicotinamide adenine dinucleotides (NAD^+^ and NADH, respectively) using a bioluminescence Luciferin-based assay (NAD/NADH-Glo™; Promega Corporation, Madison, WI, USA), according to the manufacturer instructions. Briefly, the *P. aeruginosa* biofilm was developed on the surface of the stainless-steel coupons as described earlier using LB broth, supplemented with or without acetoin (2 mM), and the coupons were incubated statically at 37°C for 24h under oxygen-depleted conditions. Biofilm and PLST cells were harvested as described earlier and the intracellular NAD^+^ and NADH were separately determined by measuring the amount of luminescence produced using a luminescence plate reader (Wallac Victor 3; PerkinElmer Life and Analytical Sciences, Shelton, CT, USA).

### 2.8 Metabolomic and statistical data analyses

#### 2.8.1 Metabolomics data analysis

Raw LC-MS spectral data were subjected to a sequence of analytical steps including raw data processing, data integrity check, data filtering, and normalization before implementing a panel of statistical models. Correlation analysis (using Pearson coefficient), principal component analysis (PCA), and partial least square discriminant analysis (PLSDA) were used to cluster biofilm and planktonic metabolomes and infer metabolomic differences among biofilm and planktonic cells at *p <* 0.05. Significant differences in metabolites’ abundance among different groups were determined using analysis of variance (ANOVA) with significance at *p <* 0.05. Additional metabolomic functional analysis was conducted using Metabolite Set Enrichment Analysis (MSEA) and Kyoto Encyclopedia of Genes and Genomes (KEGG) database to determine the metabolic pathways enriched in biofilm and planktonic cells. *P. aeruginosa* metabolome database (PAMDB, http://pseudomonas.umaryland.edu) (Huang et al., 2018) was used to infer identity of metabolites enriched in biofilm or planktonic cells. Statistical analyses, metabolite box plots, and heat map visualizations were performed in MetaboAnalyst software (Pang et al., 2021). Metabolites that are highly enriched in biofilm cells compared to planktonic cells, were analyzed using “Biomarker analysis” module in MetaboAnalyst pipeline and data were normalized to sample median and autoscaled by mean-centering and dividing each metabolite normalized abundance by its standard deviation. Autoscaling performed well in data standardization and enhanced comparability of metabolite profiles (van den Berg et al., 2006).

#### 2.8.2 *In vitro* biofilm data analysis

Data of biofilm biomass from *in vitro* assays were presented as the mean (± standard deviation derived) from two biological repeats, with each replicate conducted in duplicate, at least. A statistical software (GraphPad Prism 9.0.0; GraphPad software, San Diego, CA, USA) was used to perform ANOVA with Tukey pairwise comparisons to identify significant differences between treatment groups, with significance at *p <* 0.05.

## 3 Results

### 3.1 Untargeted metabolomic analysis reveals a clear distinction between *P. aeruginosa* biofilm and planktonic cells

**Figure 1:**
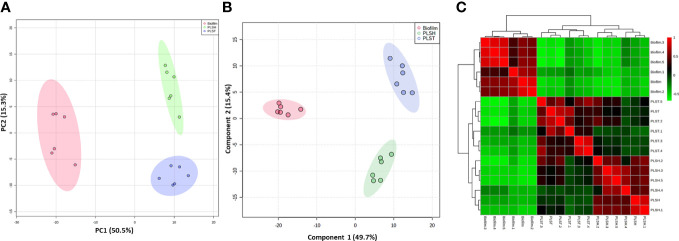
Clustering of metabolomic data showing unique metabolic differences between biofilm and two planktonic phenotypes, namely planktonic cells grown under static (PLST) or shaking (PLSH) conditions. Two-dimensional score plot from (panel **A**) principal component analyses (PCA), and (panel **B**) partial least squares discriminant analysis (PLS-DA) of biofilm (red), PLST (blue) and PLSH (green) samples based on the quantity of identified metabolites (n=394) are displayed. The heatmap (panel **C**) displayed hierarchical clustering of biofilm and planktonic samples based on Pearson correlation analysis. Color scale (panel **C**) shows the correlation coefficient value with positive and negative correlations are shown as red and green, respectively.

The metabolic differences between *P. aeruginosa* planktonic cells grown under either static (oxygen-depleted) or shaking (aerobic) conditions (PLST and PLSH, respectively) and the hypoxic biofilms cells were revealed by the untargeted metabolomics using LC-MS. The masses and concentration (measured in relative intensity units) of the metabolites were analyzed using the MetaboAnalyst pipeline. The analysis revealed a total of 394 metabolites in all samples (biofilm and planktonic cells); among these, 324 metabolites were significantly (*p <* 0.05) different in quantity among the three groups (biofilm, PLST, and PLSH) as determined by ANOVA (**Supplementary Presentation 1**). The quantitative changes in the identified metabolites, among the three groups, were assessed using PCA as an unsupervised multivariate statistical approach (**Figure 1A**). The PCA analysis revealed that samples within each group were tightly clustered, and the three phenotypic groups were well-separated, indicating distinct metabolic differences between them. When additional multivariate analysis using a supervised method, PLSDA, was conducted on the metabolomic data, the three groups were separated into well-defined clusters without overlap and within group variation was notably small (**Figure 1B**). Component “1” in PLSDA represented 49.7% of the data and confirmed the separation between the biofilm and planktonic groups.

A correlation analysis was conducted to assess the relationships between the metabolomic profiles across the distinct phenotypic groups. The resulting heatmap (**Figure 1C**) showed a clear demarcation between the biofilm group and the two planktonic groups, elucidating a prominent variance in their metabolomic landscapes. The biofilm group distinctly aggregated into a singular cluster, showcasing pronounced differentiation from the planktonic counterparts, which exhibited a closer proximity to each other, by being aggregated into a primary cluster that harbored two differentiated sub-clusters. The obvious segregation between the biofilm and planktonic clusters underscored a significant metabolic disparity and indicated a divergent metabolic phenotype of the biofilm (**Figure 1C**). Collectively, these integrative analyses encompassing PCA, PLSDA, coupled with the correlation assessment, indicated the overall well-separation and metabolic discrimination among the three phenotypic groups, signifying a scenario where the biofilm metabolome is distinct, and the planktonic phenotypes share closer metabolic features with each other, compared to the biofilm phenotype.

### 3.2 Metabolites significantly differing between biofilm and planktonic groups

**Figure 2:**
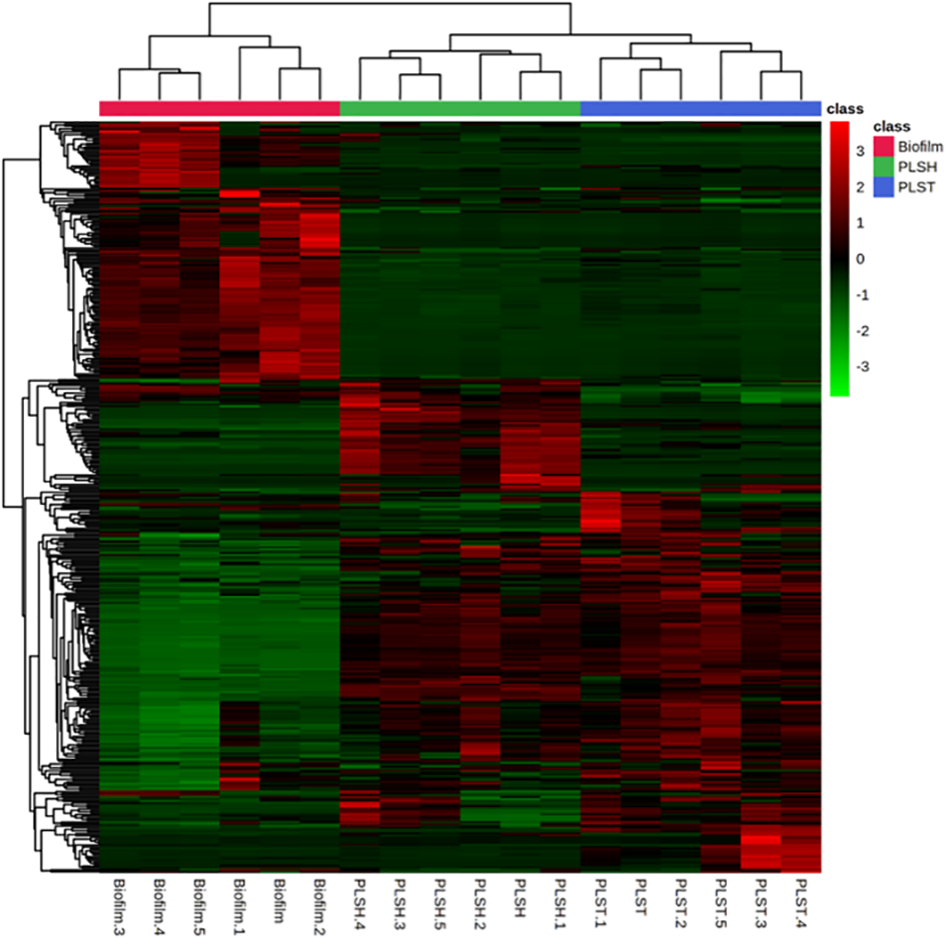
A heatmap showing hierarchical clustering of biofilm and planktonic samples into distinct cohorts using the Euclidean distance and averages of normalized quantities of the identified metabolites (n=394). The color scale shows the normalized and autoscaled quantity of metabolites, the quantity of which were normalized to the sample median. Green indicates metabolites with abundance below the mean across all samples, signifying lower abundance, while red signifies those with abundance above the mean, indicating higher abundance.

**Figure 3:**
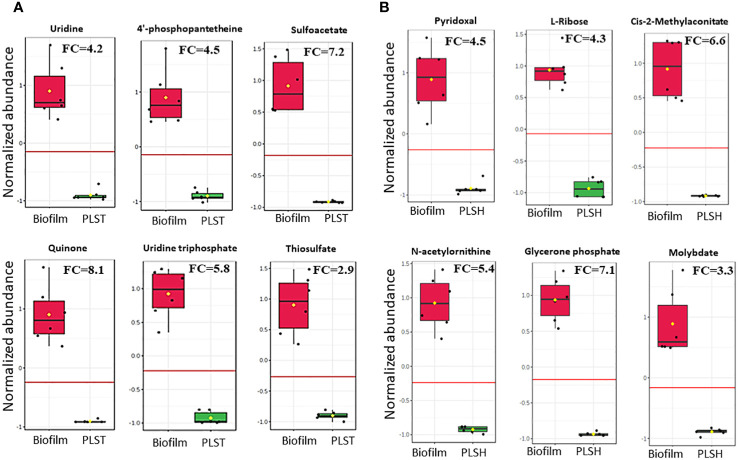
Changes in the quantity of top selected metabolites in biofilm cells and planktonic cells grown under static (PLST, panel **A**) or shaking (PLSH, panel **B**) conditions. The box plot represents normalized metabolite quantities in Biofilm (red) and PLST or PLSH (green) cells. The boxes represent upper and lower quartiles, black circles represent independent metabolite quantity values of each sample, black bars within the boxes indicate the median, yellow diamonds represent the mean value and whiskers extend to represent the overall range (minimum and maximum values) of metabolites quantities. An emphasized horizontal red line serves as a reference, marking the optimal cutoff between the two groups. Metabolite abundances were normalized to sample median and auto-scaled (mean-centered and divided by standard deviation for each metabolite). “FC” indicates fold change of each metabolite in biofilm compared to PLST or PLSH cells.

An intuitive visualization of the relative abundance of metabolites (n=394) among samples of the three groups (biofilm, PLSH, and PLST) is presented in **Figure 2**. Hierarchical clustering of the metabolites revealed a singular cluster showing highly abundant metabolites (nearly 120 metabolites) in the biofilm group compared to the two planktonic groups. Most of the remaining metabolites were highly abundant across the two planktonic groups compared to the biofilm group and were aggregated into large clusters. Based on the clustering analysis of all metabolites, the biofilm metabolome contained a significant metabolite set which are distinguishable from the planktonic phenotypes (**Figures 2**, **3**).

For the discovery of metabolites which can distinguish between the biofilm group and the two planktonic groups, a biomarker statistical analysis was performed using MetaboAnalyst pipeline and the top ranked metabolites (based on low *p* values) were selected. The efficacy of a biomarker (*i.e.*, metabolite) is further determined by assessing for true positive rate (sensitivity) and false positive rate (specificity) using the biomarker analysis tool in MetaboAnalyst. The fold change and *p* values of biofilm metabolites, determined by the biomarker analysis, compared to PLSH and PLST are shown in **Supplementary Datasheet 1**. Compared to PLST, the biofilm group displayed 121 metabolites with fold change (FC) more than 2, among which 112 had *p <* 0.05. Of the top ranked metabolites (**Figure 3A**) based on their significance (*p* value), ability to determine the metabolite identity, fold change (at least > 2), sensitivity, and specificity analyses, uridine (FC=4.2; *p*= 5.1×10^-6^), 4-phosphopantetheine (FC=4.5, *p* = 6.7×10^-6^), uridine triphosphate FC=5.8, *p*= 3.5×10^-7^), thiosulfate (FC= 2.9, *p*= 4.3×10^-6^), sulfoacetate (FC=7.2, *p*=1.4×10^-6^), and quinone (FC=8.1, *p*=4.1×10^-6^) were identified as a subset of biomarker metabolites that distinguishes biofilm from PLST cells.

Compared to PLSH, the biomarker analysis revealed 129 metabolites that were highly increased (FC > 2) in the biofilm group, and among those, 109 metabolites had *p <* 0.05. Among the top ranked biomarker metabolites (**Figure 3B**); Cis-2-methylaconitate (FC=6.6, *p*= 1.1×10^-6^), L-ribose (FC=4.3, *p*=5.3×10^-8^), N-acetylornithine (FC=5.4, *p*=4.2x10^-7^), glycerone phosphate (FC=7.1, *p*=3.8×10^-8^), pyridoxal (FC=4.5, *p*=1.2×10^-5^), and molybdate (FC=3.3, *p*=1.3×10^-5^) were identified. Moreover, the biomarker analysis revealed that there were about 319 metabolites overlapping between the two groups, biofilm compared to PLST or PLSH.

### 3.3 Metabolic pathways that were significantly enriched in *P. aeruginosa* biofilm

**Figure 4:**
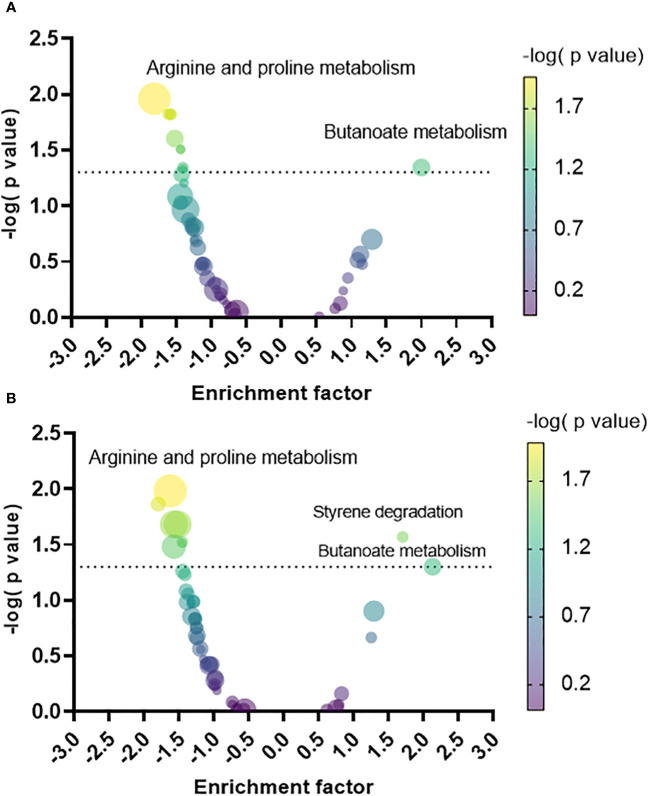
Volcano plot of the metabolic pathways that increased or decreased in the biofilm relative to planktonic cells grown under static (PLST, panel **A**) or shaking conditions (PLSH, panel **B**). Each pathway was represented by a dot; the size and color of each dot indicate the number of metabolites identified within that pathway (the smallest and the largest dots correspond to n= 2 and n=23 metabolites, respectively), and the negative logarithm of the *P* value, respectively. The dotted line indicates the minimum threshold of significance (-log_10_ of *p* = 0.05). The top pathway hits that increased or decreased in the biofilm were labelled in both panels.

A pathway enrichment analysis was conducted by employing MSEA to map the metabolites to their metabolic pathways in *P. aeruginosa* as defined in the KEGG database. The enrichment factor (EF) was calculated for each pathway based on the relative quantity of the identified metabolites in the biofilm versus the planktonic phenotypes, facilitating the discernment of what metabolic pathways were predominantly upregulated or downregulated in the biofilm phenotype relative to each planktonic phenotype. Compared to PLST (**Figure 4A**), the biofilm phenotype exhibited a significant upregulation in butanoate metabolism (EF = 1.9, *p*= 0.04). Conversely, the biofilm manifested a notable downregulation in several pathways, including arginine and proline metabolism (EF= -1.8, *p*=0.01), glycolysis and gluconeogenesis (EF= -1.5, *p*= 0.01), galactose metabolism (EF= -1.6, *p*=0.01), D-glutamine and D-glutamate metabolism (EF= -1.5, *p*=0.01), arginine biosynthesis (EF= -1.5, *p*= 0.02), fructose and mannose metabolism (EF= -1.4, *p*= 0.03), benzoate degradation (EF= -1.4, *p*=0.04), and taurine and hypotaurine metabolism (EF= -1.4, *p*= 0.04). When compared to the PLSH phenotype (**Figure 4B**), the biofilm phenotype displayed a distinct upregulation in the butanoate metabolism (EF= 2.1, *p*=0.05) and styrene degradation (EF= 1.7, *p*=0.02). Conversely, biofilm showed a substantial decrease in several pathways; these include arginine and proline metabolism (EF= -1.6, *p*=0.01), sulfur metabolism (EF= -1.8, *p*= 0.01), cysteine and methionine metabolism (EF= -1.5, *p*= 0.02), aminoacyl-tRNA biosynthesis (EF= -1.5, *p*= 0.02), D-glutamine and D-glutamate metabolism (EF= -1.4, *p*=0.02), taurine and hypotaurine metabolism (EF= -1.4, *p*= 0.03), and glycine, serine and threonine metabolism (EF= -1.5, *p*=0.03).

### 3.4 Butanoate metabolism increases in *P. aeruginosa* biofilm

**Figure 5:**
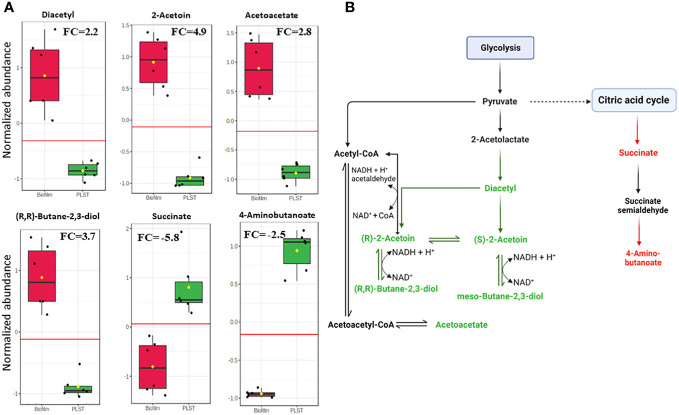
Quantitative metabolomic profiling of key metabolites in the butanoate pathway in biofilm and planktonic *P. aeruginosa* grown under static conditions (PLST). The box plots (panel **A**) represent normalized metabolite quantities in biofilm (red) and PLST (green) cells. The boxes represent upper and lower quartiles, black circles represent independent metabolite quantity values of each sample, black bars within the boxes indicate median, yellow diamonds represent the mean value and whiskers extend to represent the overall range (minimum and maximum values) of metabolites quantities. An emphasized horizontal red line serves as a reference, marking the optimal cutoff between the two groups. Metabolite abundances were normalized to sample median and auto-scaled (mean-centered and divided by standard deviation for each metabolite). “FC” indicates fold change of each metabolite in biofilm compared to PLST cells. A metabolic pathway (panel **B**) illustrating the metabolites of butanoate metabolism branch that increased (depicted as green) or decreased (depicted as red) in biofilm cells compared to planktonic cells grown under static conditions.

As just described, butanoate metabolism was a distinctive pathway of the biofilm phenotype compared to both planktonic phenotypes, PLST and PLSH. In the current study, we prioritized the comparison of butanoate metabolism in the biofilm to PLST phenotype due to the existing knowledge gap in understanding the PLST phenotype. The butanoate metabolism pathway exhibited a significant increase in the biofilm phenotype, as evident from the variation in the levels of specific butanoate metabolites (**Figure 5A**). Remarkable increases were observed in the levels of diacetyl (FC=2.2, *p*=9.6×10^-5^), (S)-/(R)-2-acetoin (FC=4.9, *p*=1.5×10^-6^), (R,R)-butane-2,3-diol (FC=3.8, *p*=1.6×10^-5^), and acetoacetate (FC=2.8, *p*=8.7×10^-6^). In contrast, two metabolites of this pathway relatively decreased in the biofilm; there are succinate (FC= -5.8, *p*=5.9×10^-4^) and 4-aminobutanoate (FC= -2.5, *p*=9.4×10^-9^). Changes in the abundance of these metabolites showed similar trends in the biofilm, compared to PLSH phenotype (**Supplementary Presentation 2**). These findings highlight the key role of butanoate metabolism in *P. aeruginosa* biofilm formation. **Figure 5B** illustrates the distinct branch of the butanoate metabolic pathway that was enriched in the biofilm. Significant variations in metabolite quantities in the biofilm relative to the PLST phenotype were indicated by distinct colors (**Figure 5B**).

### 3.5 Exogenous acetoin increased biofilm formation in *P. aeruginosa*

**Figure 6:**
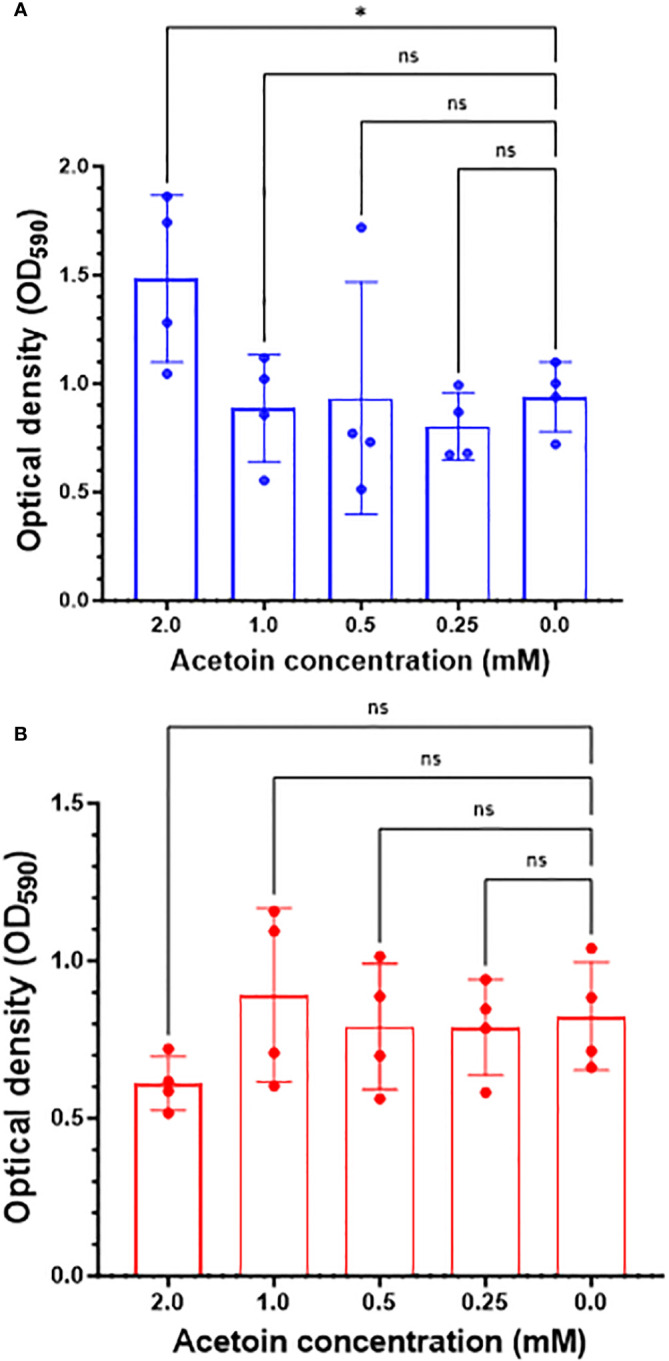
Changes in *Pseudomonas aeruginosa* biofilm mass in response to acetoin supplementation at different concentrations (0.25-2.00 mM) during biofilm formation at the early stage (blue bars, panel **A**) or 8-hour post attachment (red bars, panel **B**). Values are represented as mean ± standard deviation. Asterisks denote statistical significance (*p < 0.05) whereas “ns” indicates nonsignificant.

We examined the role of exogenous acetoin, a central metabolite in the butanoate metabolic pathway, in augmenting biofilm mass and structure at two distinct developmental stages: initial biofilm formation and 8-hour post attachment (mature phase). As shown in **Figure 6A**, the addition of acetoin (2 mM) significantly elevated the biofilm mass (*p <* 0.05) during the initial biofilm formation compared to the other tested concentrations or the untreated control. However, acetoin supplementation at the tested concentrations during the biofilm maturation stage (**Figure 6B**) did not yield a significant increase in biofilm mass (*p* > 0.05) compared to the untreated control. These findings underscore the potent effect of acetoin (2 mM) in increasing the biofilm mass at the early developmental stage.

**Figure 7:**
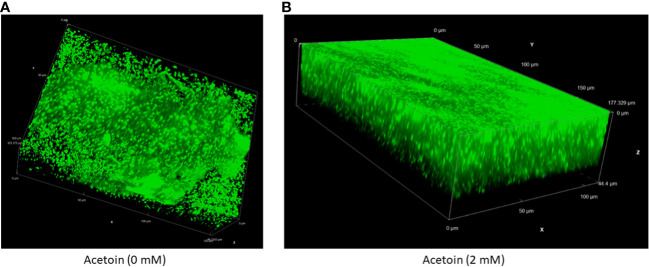
Live cell fluorescence microscopy of an inverted three-dimensional biofilm of *Pseudomonas aeruginosa* developed without (panel **A**) or with (panel **B**) acetoin at 2 mM for 24 (h). The biofilms were stained with a nucleic acid fluorescent stain and analyzed at 60X magnification.

To assess the influence of acetoin on biofilm architecture, live-cell confocal laser scanning microscopy was employed to characterize the 3-dimentional structure and thickness of biofilms grown in the presence or absence of 2 mM acetoin. As illustrated in **Figure 7** and **Supplementary Video 1**, biofilms exposed to acetoin exhibited increased compactness and thickness. This finding highlights the significant role of acetoin in enhancing biofilm structural integrity; this may pave the way for in-depth investigations into the mechanistic basis of this observation.

### 3.6 Exogenous acetoin altered the redox state in the biofilm cells

**Figure 8:**
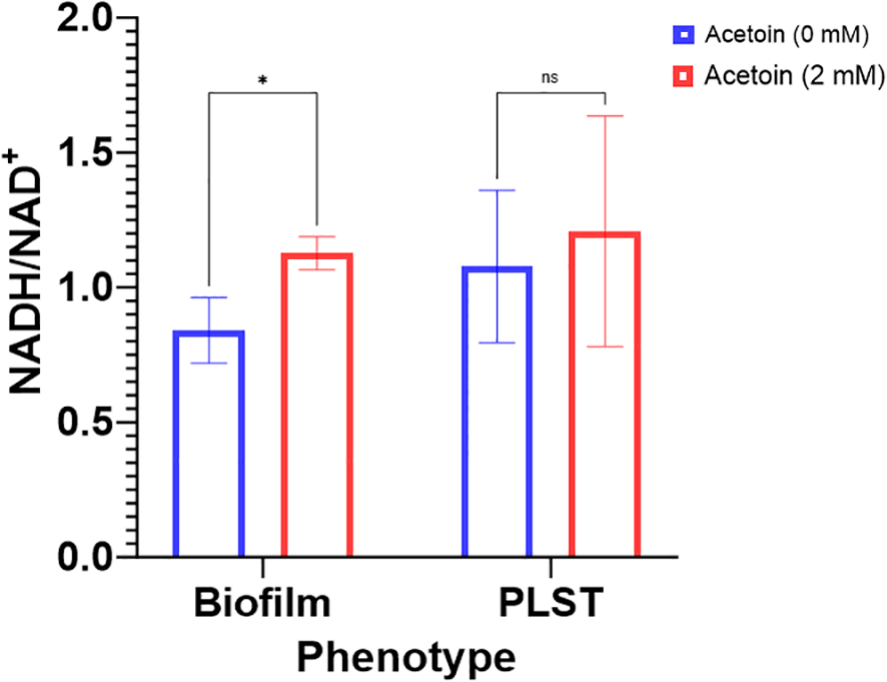
Changes in intracellular levels of NADH/NAD^+^ ratio in *Pseudomonas aeruginosa* biofilm and planktonic cells grown under static conditions (PLST) and incubated at oxygen-depleted conditions for 24h in presence or absence of acetoin at 2mM. Data were presented as mean ± standard deviation and statistical significance was determined at *p <* 0.05 while “ns” denotes for nonsignificant. * denotes p < 0.05.

The enrichment of the butanoate metabolic pathway in *P. aeruginosa* biofilm, relative to planktonic cells, led us to probe the pathway influence on biofilm formation. Supplementation of acetoin during biofilm growth on stainless steel coupons led to elevated concentrations of both NADH and NAD^+^. Notably, the NADH/NAD^+^ ratio in the acetoin-treated biofilm was significantly higher (*p <* 0.05) than in the untreated biofilm, as shown in **Figure 8**. These findings suggest that acetoin, a pivotal metabolite in the butanoate pathway, facilitates the accumulation of NADH, which is channeled towards ATP synthesis. The biofilm-associated NADH/NAD^+^ ratio of 1.12 in the presence of acetoin, indicates the effect of acetoin on sustaining the redox balance within the biofilm cells. Conversely, acetoin’s impact on NADH/NAD^+^ levels was insignificant in PLST cells when compared to their untreated counterparts (**Figure 8**).

## 4 Discussion

Biofilm formation by opportunistic pathogens, such as *P. aeruginosa*, represents a significant clinical challenge. This is particularly evident when examining the role of biofilms in diseases such as cystic fibrosis and joint infections (Rossi et al., 2020; Leggett et al., 2022a). There are increasing clinical concerns about biofilms in environments such as cystic fibrosis airways where oxygen levels might be considerably low. The target of the current investigation was the metabolic and physiological attributes of *P. aeruginosa* biofilm under oxygen-depleted conditions simulating the hypoxic or anoxic environments, which bacteria often encounter *in vivo* (e.g., cystic fibrosis lungs) (Yoon et al., 2002). While our study was conducted under *in vitro* conditions, the results could lay the ground for future *in vivo* explorations. Stainless steel (304) coupons were used for biofilm development due to their ability to increase biofilm biomass for several species such as *P. aeruginosa*, *Listeria monocytogenes*, and *Desulfovibrio desulfuricans* without antibacterial activities (Lopes et al., 2006; Jia et al., 2017; Papaioannou et al., 2018). However, the use of these coupons for prolonged biofilm development should be revisited since lengthy incubation (7-14 days) of *P. aeruginosa* biofilms on the surface of 304 stainless steel and under strict anaerobic conditions could cause corrosion of this metallic surface (Jia et al., 2017).

Data analysis using PCA, PLSDA, and correlation analyses, revealed the specialized and well-defined biofilm metabolism of *P. aeruginosa*, which is distinctly separated from the metabolic profile of planktonic cells. The studied planktonic cells, grown under shaking conditions (PLSH) and those under identical biofilm static conditions (PLST), provided a unique insight into the differences of their metabolic and physiological attributes compared to the biofilm-forming cells. The metabolic distinction (**Figures 1**, **2**) suggests that biofilm formation is not a mere outcome of environmental factors, but the phenotype required specialized metabolic responses. These conclusions are supported by a recent finding that showed the distinct metabolic profile of *P. aeruginosa* biofilm grown in synovial fluid, compared to the planktonic cells (Leggett et al., 2022a).

Biofilm phenotypes displayed heightened levels of metabolites which were low in abundance in planktonic cells (**Figure 3**). Among these metabolites, uridine, 4-phosphopantethene, sulfoacetate, quinone, uridine triphosphate, and thiosulfate were produced at higher levels by biofilm cells, compared to PLST cells. Pyridoxal, L-ribose, cis-2-methylaconitate, N-acetylornithine, glycerone phosphate, and molybdate accumulated to higher degrees in biofilm cells, compared to PLSH cells. The potential role of these metabolites in biofilm formation is not fully explored. Metabolites such as uridine, uridine triphosphate, and L-ribose may be involved in inducing quorum sensing (Ueda et al., 2009; Kim et al., 2020). The metabolite 4-phosphopantethene was found to accumulate in a *Pseudomonas* biofilm (Southey-Pillig et al., 2005) whereas pyridoxal was involved in vitamin B6 biosynthesis, a metabolic process that was linked to biofilm formation in several pathogens (Wicaksono et al., 2022). Molybdate was found to be required for anaerobic respiration of *P. aeruginosa* and potentially important for biofilm formation (Pederick et al., 2014). Quinone, thiosulfate, and sulfoacetate are involved in several pathways which include, but not limited to, amino acid metabolism, butanoate metabolism, glyoxylate and dicarboxylate metabolism, sulfur metabolism, and taurine and hypotaurine metabolism. However, the direct mechanisms by which all these metabolites accumulate in the biofilm remain to be determined. The practical implications of these biofilm-associated metabolites are imperative in identifying potential metabolic biomarkers for *P. aeruginosa* biofilm under *in vitro* conditions, laying a foundation for further *in vivo* confirmatory investigations to establish novel biomarker-based diagnostic tools.

Functional analysis in the current study revealed that the central metabolic pathways including glycolysis and gluconeogenesis, tricarboxylic acid cycle, and the metabolism of amino acids such as arginine, proline, glutamine, and glutamate were downregulated in the biofilm. These findings clearly indicate that the central metabolism of biofilm cells was reduced and that the biofilm cells are less metabolically active than the planktonic cells. These findings align with previous reports which support the thought that the reduced metabolic activity is a characteristic of biofilm cells (Rossi et al., 2020; Leggett et al., 2022a).

Intriguingly, the pronounced enrichment of the butanoate metabolic pathway in *P. aeruginosa* biofilm, relative to planktonic cells, was distinct (**Figure 4**) in the current study; this led us to probe its influence on biofilm formation. To investigate the role of butanoate pathway in biofilm formation, we adopted a targeted strategy of external supplementation of acetoin. In addition to being a key metabolite within this pathway, acetoin enrichment in the biofilm metabolome (**Figure 5**) warranted the investigation. A similar metabolite supplementation strategy was implemented in a previous work where cadaverine decreased in *P. aeruginosa* biofilm (Leggett et al., 2022b). When the researchers exogenously supplemented cadaverine into the microbiological medium of *P. aeruginosa*, biofilm decreased and planktonic growth increased (Leggett et al., 2022b). In the current study, acetoin was supplemented in LB broth to study its possible role in biofilm development. Acetoin was thought to be a volatile biomarker in exhaled breath of cystic fibrosis (a biofilm-related disease) patients, but this assumption still needs further investigation in large patient cohorts (Španěl et al., 2016). Besides, acetoin was thought to establish a trophic cooperation between *P. aeruginosa* and *S. aureus* in the context of cystic fibrosis lung environment, and the compound was catabolized by *P. aeruginosa* as a carbon source (Camus et al., 2020).

Supplementation of acetoin during biofilm growth on stainless-steel coupons led to elevated biofilm biomass, increased biofilm compactness and thickness, and augmented the NADH/NAD^+^ ratio (**Figures 6**–**8**). The preliminary genomic analysis of *P. aeruginosa* ATCC 9027 revealed enzymes which reversibly convert acetoin to butane-2,3-diol using butane-2,3-diol:NAD^+^ oxidoreductases and converting acetoin into acetaldehyde and acetyl-CoA by acetoin dehydrogenase (**Figure 5B**; **Supplementary Table 1**). These metabolic reactions generate NADH and NAD^+^. In that context, the NADH/NAD^+^ ratio in the acetoin-treated biofilm was notably higher (*p <* 0.05) than that in the untreated biofilm controls (**Figure 8**). This interconversion of acetoin into 2,3-butandiol and acetyl-CoA suggests recycling of NADH and NAD^+^ in *P. aeruginosa* biofilm. The NADH/NAD^+^ ratio of 1.12 in acetoin-treated biofilms implies a potential redox equilibrium within these cells, promoting their biofilm growth. Conversely, acetoin’s impact on NADH/NAD^+^ levels was insignificant in PLST cells when compared to their untreated counterparts. Results of this study suggest that acetoin, a pivotal metabolite in the butanoate pathway, augments NADH for ATP synthesis in biofilm cells and maintains cell redox homeostasis by equilibrating NADH and NAD^+^. Earlier work indicated a role for acetoin in redox balance in *Zymomonas mobilis* (Bao et al., 2023).To the best of our knowledge, the current work is the first report emphasizing acetoin’s significance in *P. aeruginosa* biofilm growth, potentially, via the butanoate metabolic pathway. The interconversion of the acetoin to 2,3 butanediol or acetoin catabolism into acetyl-CoA and acetaldehyde (**Supplementary Table 1**) is important in maintaining the intracellular redox balance.

## 5 Conclusion

Previous research rarely addressed *P. aeruginosa* biofilm metabolism under oxygen-depleted conditions or how different it is from the metabolism in the co-existing static planktonic cells. The current study supported the notion that the metabolism of *P. aeruginosa* biofilm under oxygen-depleted conditions is markedly distinct from that in planktonic phenotypes. The biofilm metabolism reduced central metabolic pathways but enriched the butanoate pathway for maintaining biofilm growth and intracellular redox homeostasis. Exogenous supplementation of acetoin, a key metabolite in the butanoate pathway, enhanced biofilm characteristics, and maintained a balanced NADH/NAD^+^ ratio. Thus, inhibiting butanoate metabolism could be a new strategy for combating *P. aeruginosa* biofilm. As our understanding of biofilm physiology and metabolism expands, pinpointing biofilm metabolic markers will be vital for devising targeted therapeutic interventions for biofilm-associated infections. Moving forward, the next phase of our research seeks to translate these insights to *in vivo* settings, ultimately aiming to refine biofilm therapeutics.

## Data availability statement

The original contributions presented in the study are included in the article's **Supplementary Material**. Further inquiries can be directed to the corresponding author.

## Author contributions

AA: Conceptualization, Data curation, Investigation, Methodology, Visualization, Writing – original draft, Writing – review & editing. AY: Conceptualization, Funding acquisition, Project administration, Supervision, Writing – review & editing.

## References

[B1] Bao W. Shen W. Peng Q. Du J. Yang S. (2023). Metabolic engineering of *Zymomonas mobilis* for acetoin production by carbon redistribution and cofactor balance. Fermentation 9, 113. doi: 10.3390/fermentation9020113

[B2] Bomberger J. M. MacEachran D. P. Coutermarsh B. A. Ye S. O’Toole G. A. Stanton B. A. (2009). Long-distance delivery of bacterial virulence factors by *Pseudomonas aeruginosa* outer membrane vesicles. PloS Pathog. 5, e1000382. doi: 10.1371/journal.ppat.1000382 19360133 PMC2661024

[B3] Botelho J. Grosso F. Peixe L. (2019). Antibiotic resistance in *Pseudomonas aeruginosa* – Mechanisms, epidemiology and evolution. Drug Resist. Updat. 44, 100640. doi: 10.1016/j.drup.2019.07.002 31492517

[B4] Camus L. Briaud P. Bastien S. Elsen S. Doléans-Jordheim A. Vandenesch F.. (2020). Trophic cooperation promotes bacterial survival of *Staphylococcus aureus* and *Pseudomonas aeruginosa* . ISME J. 14, 3093–3105. doi: 10.1038/s41396-020-00741-9 32814867 PMC7784975

[B5] Cendra M.d. M. Torrents E. (2021). *Pseudomonas aeruginosa* biofilms and their partners in crime. Biotechnol. Adv. 49, 107734. doi: 10.1016/j.bioteChadv.2021.107734 33785375

[B6] Coffey B. M. Anderson G. G. (2014). Biofilm formation in the 96-well microtiter plate. Methods Mol. Biol. 1149, 631–641. doi: 10.1007/978-1-4939-0473-0_48 24818938

[B7] Crabbé A. Jensen P.Ø. Bjarnsholt T. Coenye T. (2019). Antimicrobial tolerance and metabolic adaptations in microbial biofilms. Trends Microbiol. 27, 850–863. doi: 10.1016/j.tim.2019.05.003 31178124

[B8] Di Onofrio V. Gesuele R. Maione A. Liguori G. Liguori R. Guida M.. (2019). Prevention of *Pseudomonas aeruginosa* biofilm formation on soft contact lenses by allium sativum fermented extract (BGE) and cannabinol oil extract (CBD). Antibiotics 8, 258. doi: 10.3390/antibiotics8040258 31835470 PMC6963262

[B9] Huang W. Brewer L. K. Jones J. W. Nguyen A. T. Marcu A. Wishart D. S.. (2018). PAMDB: A comprehensive *Pseudomonas aeruginosa* metabolome database. Nucleic Acids Res. 46, D575–D580. doi: 10.1093/nar/gkx1061 29106626 PMC5753269

[B10] Jia R. Yang D. Xu D. Gu T. (2017). Anaerobic corrosion of 304 stainless steel caused by the *Pseudomonas aeruginosa* biofilm. Front. Microbiol. 8. doi: 10.3389/fmicb.2017.02335 PMC5712129 29230206

[B11] Jurado-Martín I. Sainz-Mejías M. McClean S. (2021). *Pseudomonas aeruginosa*: An audacious pathogen with an adaptable arsenal of virulence factors. Int. J. Mol. Sci. 22, 1–37. doi: 10.3390/IJMS22063128 PMC8003266 33803907

[B12] Kamarajan B. P. Muthusamy A. (2020). Survival strategy of *Pseudomonas aeruginosa* on the nanopillar topography of dragonfly (Pantala flavescens) wing. AMB Express 10, 85. doi: 10.1186/s13568-020-01021-7 32378141 PMC7203277

[B13] Kim E. K. Lee K. A. Hyeon D. Y. Kyung M. Jun K. Y. Seo S. H.. (2020). Bacterial nucleoside catabolism controls quorum sensing and commensal-to-pathogen transition in the *Drosophila* gut. Cell Host Microbe 27, 345–357.e6. doi: 10.1016/j.chom.2020.01.025 32078802

[B14] La Rosa R. Johansen H. K. Molin S. (2018). Convergent metabolic specialization through distinct evolutionary paths in *Pseudomonas aeruginosa* . MBio 9, e00269–e00318. doi: 10.1128/mbio.00269-18 29636437 PMC5893872

[B15] Leggett A. Li D. W. Bruschweiler-Li L. Sullivan A. Stoodley P. Brüschweiler R. (2022a). Differential metabolism between biofilm and suspended *Pseudomonas aeruginosa* cultures in bovine synovial fluid by 2D NMR-based metabolomics. Sci. Rep. 12, 17317. doi: 10.1038/s41598-022-22127-x 36243882 PMC9569359

[B16] Leggett A. Li D. W. Sindeldecker D. Staats A. Rigel N. Bruschweiler-Li L.. (2022b). Cadaverine is a switch in the lysine degradation pathway in *Pseudomonas aeruginosa* biofilm identified by untargeted metabolomics. Front. Cell. Infect. Microbiol. 12. doi: 10.3389/fcimb.2022.833269 PMC8884266 35237533

[B17] Lopes F. A. Morin P. Oliveira R. Melo L. F. (2006). Interaction of *Desulfovibrio desulfuricans* biofilms with stainless steel surface and its impact on bacterial metabolism. J. Appl. Microbiol. 101, 1087–1095. doi: 10.1111/j.1365-2672.2006.03001.x 17040232

[B18] McCarthy K. L. Paterson D. L. (2017). Increased risk of death with recurrent *Pseudomonas aeruginosa* bacteremia. Diagn. Microbiol. Infect. Dis. 88, 152–157. doi: 10.1016/j.diagmicrobio.2017.03.001 28366610

[B19] Pang Z. Chong J. Zhou G. De Lima Morais D. A. Chang L. Barrette M.. (2021). MetaboAnalyst 5.0: Narrowing the gap between raw spectra and functional insights. Nucleic Acids Res. 49, W388–W396. doi: 10.1093/nar/gkab382 34019663 PMC8265181

[B20] Papaioannou E. Giaouris E. D. Berillis P. Boziaris I. S. (2018). Dynamics of biofilm formation by *Listeria monocytogenes* on stainless steel under mono-species and mixed-culture simulated fish processing conditions and chemical disinfection challenges. Int. J. Food Microbiol. 267, 9–19. doi: 10.1016/j.ijfoodmicro.2017.12.020 29275280

[B21] Pederick V. G. Eijkelkamp B. A. Ween M. P. Begg S. L. Paton J. C. McDevitt C. A. (2014). Acquisition and role of molybdate in *Pseudomonas aeruginosa* . Appl. Environ. Microbiol. 80, 6843–6852. doi: 10.1128/AEM.02465-14 25172858 PMC4249025

[B22] Qin S. Xiao W. Zhou C. Pu Q. Deng X. Lan L.. (2022). *Pseudomonas aeruginosa*: pathogenesis, virulence factors, antibiotic resistance, interaction with host, technology advances and emerging therapeutics. Signal Transduction Targeting Ther. 7, 199. doi: 10.1038/s41392-022-01056-1 PMC9233671 35752612

[B23] Rossi E. La Rosa R. Bartell J. A. Marvig R. L. Haagensen J. A. J. Sommer L. M.. (2020). *Pseudomonas aeruginosa* adaptation and evolution in patients with cystic fibrosis. Nat. Rev. Microbiol. 2020 195 19, 331–342. doi: 10.1038/s41579-020-00477-5 33214718

[B24] Southey-Pillig C. J. Davies D. G. Sauer K. (2005). Characterization of temporal protein production in *Pseudomonas aeruginosa* biofilms. J. Bacteriol. 187, 8114–8126. doi: 10.1128/JB.187.23.8114-8126.2005 16291684 PMC1291272

[B25] Španěl P. Sovová K. Dryahina K. Doušová T. Dřevínek P. Smith D. (2016). Do linear logistic model analyses of volatile biomarkers in exhaled breath of cystic fibrosis patients reliably indicate *Pseudomonas aeruginosa* infection? J. Breath Res. 10, 36013. doi: 10.1088/1752-7155/10/3/036013 27532768

[B26] Ueda A. Attila C. Whiteley M. Wood T. K. (2009). Uracil influences quorum sensing and biofilm formation in *Pseudomonas aeruginosa* and fluorouracil is an antagonist. Microb. Biotechnol. 2, 62–74. doi: 10.1111/j.1751-7915.2008.00060.x 21261882 PMC3815422

[B27] van den Berg R. A. Hoefsloot H. C. J. Westerhuis J. A. Smilde A. K. van der Werf M. J. (2006). Centering, scaling, and transformations: Improving the biological information content of metabolomics data. BMC Genomics 7, 1–15. doi: 10.1186/1471-2164-7-142 16762068 PMC1534033

[B28] Wicaksono W. A. Erschen S. Krause R. Müller H. Cernava T. Berg G. (2022). Enhanced survival of multi-species biofilms under stress is promoted by low-abundant but antimicrobial-resistant keystone species. J. Hazard. Mater. 422, 126836. doi: 10.1016/j.jhazmat.2021.126836 34403940

[B29] Wu X. (2019). *In vivo* proteome of *Pseudomonas aeruginosa* in airways of cystic fibrosis patients. J. Proteome. Res. 18, 2601–2612. doi: 10.1021/acs.jproteome.9b00122 31060355 PMC6750005

[B30] Yoon S. S. Hennigan R. F. Hilliard G. M. Ochsner U. A. Parvatiyar K. Kamani M. C.. (2002). *Pseudomonas aeruginosa* anaerobic respiration in biofilms: Relationships to cystic fibrosis pathogenesis. Dev. Cell 3, 593–603. doi: 10.1016/S1534-5807(02)00295-2 12408810

[B31] Zhou G. Peng H. Wang Y. S. Huang X. M. Xie X. B. Shi Q. S. (2019). Enhanced synergistic effects of xylitol and isothiazolones for inhibition of initial biofilm formation by *Pseudomonas aeruginosa* ATCC 9027 and *Staphylococcus aureus* ATCC 6538. J. Oral. Sci. 61, 255–263. doi: 10.2334/josnusd.18-0102 31217374

