# Supplementary material for: Untargeted metabolomics unveiled the role of butanoate metabolism in the development of Pseudomonas aeruginosa hypoxic biofilm
Source: Front Cell Infect Microbiol. 2024 Feb 16;14:1346813. doi: 10.3389/fcimb.2024.1346813 (PMC10904581; doi:10.3389/fcimb.2024.1346813)
Supplement: Supplementary file 2 [file Presentation_1.pptx]

## Slide 1
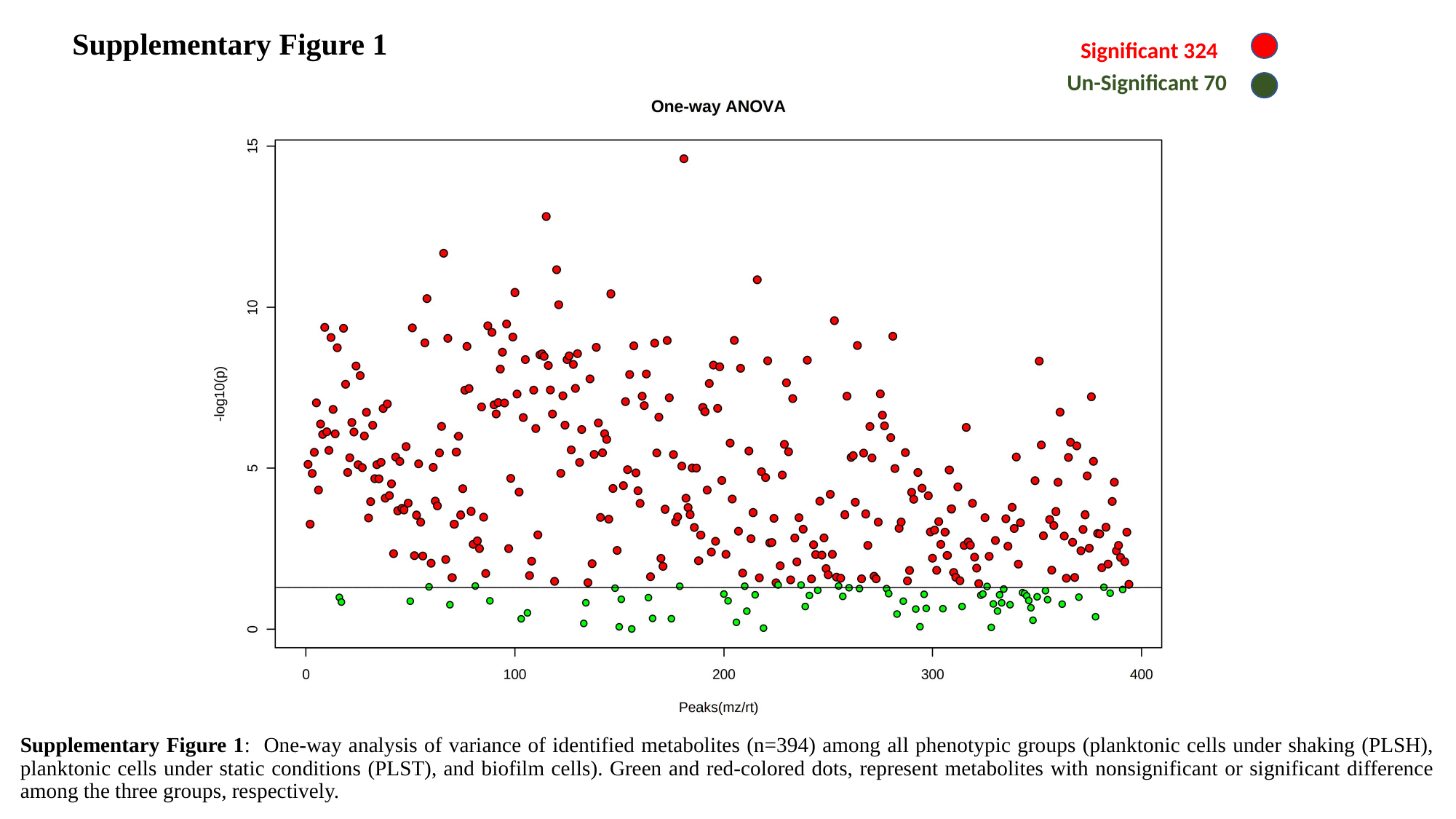

Supplementary Figure 1
Significant 324
Un-Significant 70
# Supplementary Figure 1: One-way analysis of variance of identified metabolites (n=394) among all phenotypic groups (planktonic cells under shaking (PLSH), planktonic cells under static conditions (PLST), and biofilm cells). Green and red-colored dots, represent metabolites with nonsignificant or significant difference among the three groups, respectively.
