# Supplementary material for: Untargeted metabolomics unveiled the role of butanoate metabolism in the development of Pseudomonas aeruginosa hypoxic biofilm
Source: Front Cell Infect Microbiol. 2024 Feb 16;14:1346813. doi: 10.3389/fcimb.2024.1346813 (PMC10904581; doi:10.3389/fcimb.2024.1346813)
Supplement: Supplementary file 3 [file Presentation_2.pptx]

## Slide 1
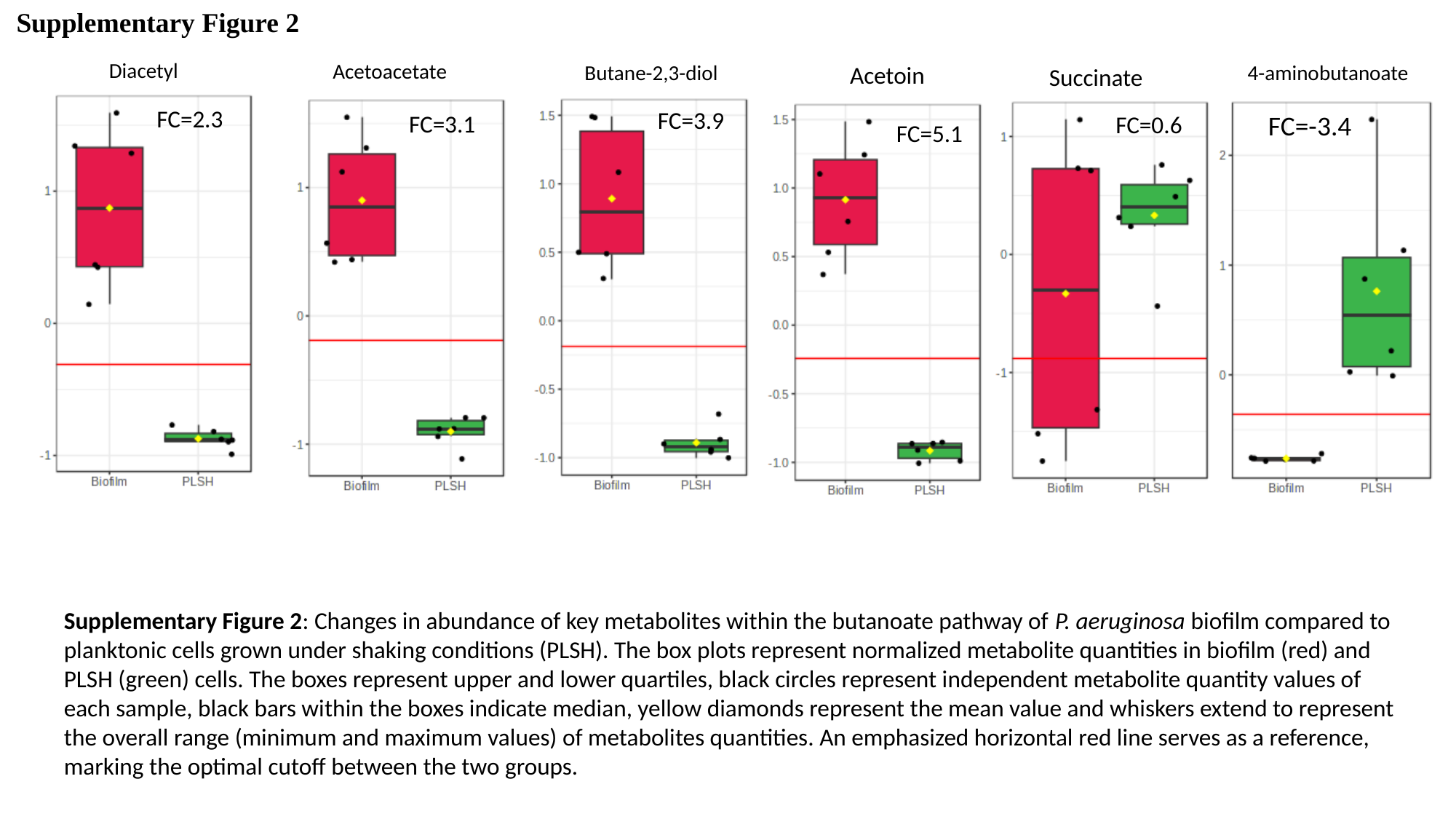

Supplementary Figure 2
Diacetyl
FC=2.3
Acetoacetate
FC=3.1
Butane-2,3-diol
FC=3.9
4-aminobutanoate
FC=-3.4
Acetoin
FC=5.1
Succinate
FC=0.6
Supplementary Figure 2: Changes in abundance of key metabolites within the butanoate pathway of P. aeruginosa biofilm compared to planktonic cells grown under shaking conditions (PLSH). The box plots represent normalized metabolite quantities in biofilm (red) and PLSH (green) cells. The boxes represent upper and lower quartiles, black circles represent independent metabolite quantity values of each sample, black bars within the boxes indicate median, yellow diamonds represent the mean value and whiskers extend to represent the overall range (minimum and maximum values) of metabolites quantities. An emphasized horizontal red line serves as a reference, marking the optimal cutoff between the two groups.
