# Supplementary material for: Untargeted metabolomics unveiled the role of butanoate metabolism in the development of Pseudomonas aeruginosa hypoxic biofilm
Source: Front Cell Infect Microbiol. 2024 Feb 16;14:1346813. doi: 10.3389/fcimb.2024.1346813 (PMC10904581; doi:10.3389/fcimb.2024.1346813)
Supplement: Supplementary file 4 [file Table_1.docx]

Acetoin metabolism in *P. aeruginosa* ATCC 9027 according to a metabolic model constructed by ModelSEED2 pipeline in KBase:

1. By the enzyme S,S-Butane-2,3-diol:NAD^+^ oxidoreductase

NAD + (S,S)-2,3-Butanediol <=> NADH + H^+^ + (S)-Acetoin

1. By enzyme (R,R)-Butane-2,3-diol:NAD+ oxidoreductase

NAD + (R,R)-2,3-Butanediol <=> NADH + H^+^ + (R)-Acetoin

1. By the enzyme acetyl-CoA:acetoin O-acetyltransferase

NAD + CoA + Acetoin <=> NADH + Acetyl-CoA + H^+^ + Acetaldehyde
